# RETRACTION: PDE5A Suppresses Proteasome Activity Leading to Insulin Resistance in C2C12 Myotubes

**DOI:** 10.1155/ije/9851367

**Published:** 2026-07-14

**Authors:** International Journal of Endocrinology

RETRACTION: W. Liu, X. Tian, T. Wu, L. Liu, Y. Guo, and C. Wang, “PDE5A Suppresses Proteasome Activity Leading to Insulin Resistance in C2C12 Myotubes,” *International Journal of Endocrinology* 2019, no. 1 (2019): 3054820, https://doi.org/10.1155/2019/3054820.

The above article, published online on 15 January 2019 in Wiley Online Library (https://wileyonlinelibrary.com), has been retracted by agreement between the authors; the journal Editor‐in‐Chief, Zhongjian Xie; and John Wiley & Sons Ltd. The retraction has been agreed after an investigation into concerns raised via PubPeer [1].

Specifically, unexpected similarities were noted between figures in this article and figures in previously published articles from some of the same authors:

• Figure 1(a) and Figure 1D in [2].
• Figure 1(a) and Figure 4D in [3].
• Figure 1(a) and Figure 5A in [4].
• Figure 4 (b) and Figure 5A & 6E in [5].

Furthermore, unexpected similarities between a number of western blots in the article were found with a number of additional publications by some of the same authors [6–8].

The authors claim that these similarities were due to inappropriate image re‐use arising from irregular laboratory data management, and insufficient presubmission figure inspection. The authors could not provide their raw data and requested that the article be retracted. The editor and publisher have lost confidence in the results and conclusions reported and therefore agree with retraction of the article.
